# Unveiling the influence of circulating immune cells count on type 1 diabetes: Insight from bidirectional Mendelian randomization

**DOI:** 10.1097/MD.0000000000039842

**Published:** 2024-09-27

**Authors:** Jia Luo, Jing Wang, Yukun Xiang, Ningning Wang, Xin Zhao, GengYan Liu, Lihui Liu, Haoxiao Chang

**Affiliations:** a Changsha Blood Center, Changsha, Hunan Province, China; b China National Clinical Research Center for Neurological Diseases, Beijing Tiantan Hospital, Capital Medical University, Beijing, China; c Department of Orthopedics, The Third Xiangya Hospital, Central South University, Changsha, Hunan Province, China.

**Keywords:** casual relationship, circulating immune cell, genome-wide association study, Mendelian randomization, type 1 diabetes

## Abstract

Observational studies have demonstrated an association between circulating immune cell and type 1 diabetes (T1D) risk. However, it is unknown whether this relationship is causal. Herein, we adopted a 2-sample bidirectional Mendelian randomization study to figure out whether circulating immune cell profiles causally impact T1D liability. Summary statistical data were obtained from genome-wide association study (GWAS) to investigate the causal relationship between white cell (WBC) count, 5 specific WBC count, and lymphocyte subtypes cell count and T1D risk. After false discovery rate (FDR) correction, the results indicated that lower lymphocyte cell count (odds ratio [OR] per 1 standard deviation [SD] decrease = 0.746, 95% confidence interval (CI): 0.673–0.828, *P*_FDR_ = 0.036), and basophil cell count (OR per 1 SD decrease = 0.808, 95% CI: 0.700–0.932, *P*_FDR_ = 0.010) were causally associated with T1D susceptibility. However, the absolute count of WBC, monocyte, neutrophil, eosinophil, and lymphocyte subtypes cell had no statistically significant effect on T1D risk. Taken together, this study indicates suggestive association between circulating immune cell count and T1D. Moreover, lower numbers of circulating lymphocyte and basophil cell were associated with the increased risk of T1D, which confirmed the immunity predisposition for T1D.

## 1. Introduction

Type 1 diabetes (T1D) is a prevalent chronic autoimmune disease that affects approximately 30 million individuals, accounting for 10% of all diabetes cases.^[1]^ Incidence peaks in puberty and early adulthood, but onset can occur at any age. In Europe, the average annual increase in incidence rate was approximately 3-4% over past decades.^[2,3]^ T1D and its complications continue to pose a growing burden worldwide.

The pathogenesis of T1D involves the autoimmune destruction of pancreatic β-cells, resulting in an absolute insulin deficiency and resultant hyperglycemia.^[4]^ Over the past decades, a number of studies have implied that circulating immune cell functions have changed, playing crucial roles in the initiation and progression of T1D.^[5]^ Although the main effectors of β-cell impairment and death are T cells, leukocytes of the innate arm of the immune system are active in the early part of the inflammation process inside the pancreas.^[6]^

Several Mendelian randomization (MR) analyses have found a direct causal relationship between circulating immune cells count and the occurrence of some diseases, including multiple sclerosis,^[7]^ severe COVID-19^[8]^ and chronic obstructive pulmonary disease.^[9]^ Previous observational study showed that the number of immune cell, such as neutrophil and lymphocyte, in patients with T1D may be associated with β cell specific autoimmunity.^[10,11]^ However, the existence of a causal link between circulating immune cell count and the onset of T1D remains unclear.

Traditional observational studies often suffer from limitations owing to confounding factors, reverse causality, and other biases, making it challenging to accurately discern the causal relationship between different peripheral blood cell count and the onset of T1D.^[12]^ MR offers a solution using genetic variations as an instrumental variables (IVs) to examine the causal association between exposure factors and outcomes.

This method randomizes the population based on the free combination of alleles during gamete formation, effectively eliminating the influence of confounding factors, reverse causality, and other biases.^[13]^ The present study used bidirectional 2-sample MR methods and single nucleotide polymorphisms (SNPs) as IVs, based on open-source genome-wide association study (GWAS) data, to examine the causal relationship between circulating immune cell count and T1D.

## 2. Methods

### 2.1. Study design

**Figure 1.**
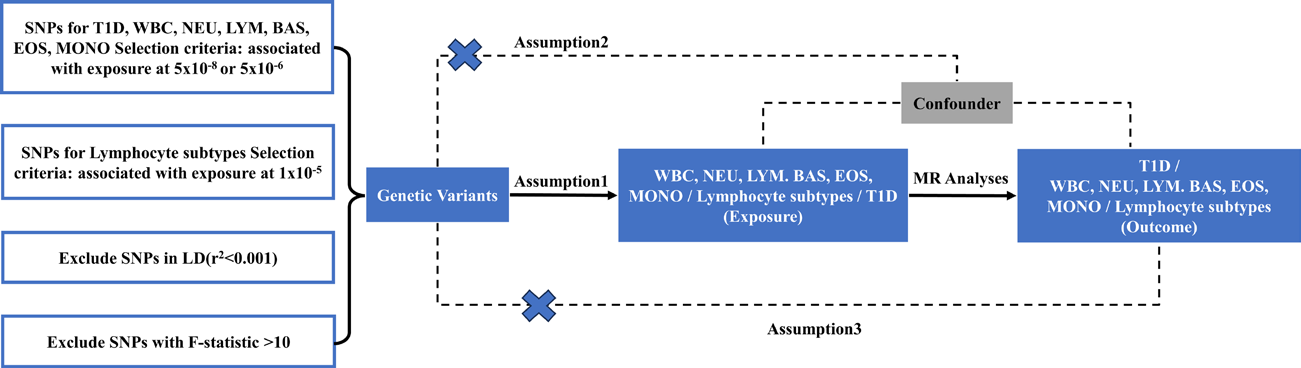
Schematic diagram for the Mendelian randomization analysis exploring effects of circulating cell count on type 1 diabetes. BAS = basophil, EOS = eosinophil, IVW = inverse variance weighted, LYM = lymphocyte, Mono = monocyte, MR = Mendelian randomization, NEU = neutrophil, SNP = single nucleotide polymorphism, WBC = white cell.

We systematically determined the causal effects of circulating immune cell and lymphocyte subtypes cell on the risk of T1D using a bidirectional 2-sample MR study. We first assessed the causal link between white cell (WBC) count and T1D, and then explored the causal relationship between 5 specific WBC count and T1D: neutrophil (NEU), basophil (BAS), eosinophil (EOS), monocyte (MON), and lymphocyte (LYM). Additionally, this study investigated the correlation between the LYM subtypes cell count and T1D. MR uses genetic variation to represent risk factors, and therefore, valid IVs in causal inference must satisfy 3 key assumptions: genetic variation have a significant association with exposure; genetic variation is not associated with possible confounders between exposure and outcome; genetic variation impact T1D solely through exposure and not via alternative routes, as illustrated in Figure 1.

### 2.2. Data sources for exposure and outcome

We sourced GWAS data pertaining to WBC, NEU, BAS, EOS, MON, and LYM cell count from the Blood Cell Consortium (BCX) (http://www.mhi-humangenetics.org), which included 563,946 European ancestry participants.^[7,14]^ GWAS summary statistics for LYM subtypes cell are publicly available from the GWAS Catalog (accession numbers from GCST0001391 to GCST0002121).^[15]^ A total of 731 immunophenotypes including absolute cell count (n = 118), median fluorescence intensities reflecting surface antigen levels (n = 389), morphological parameters (n = 32) and relative cell count (n = 192) were included.The original GWAS on immune traits was performed using data from 3757 European individuals and there was no overlapping cohorts. Approximately 22 million SNPs genotyped with high-density arrays were imputed with Sardinian sequence-based reference panel, and associations were tested after adjusting for covariates (i.e., sex, age, and age^2^).^[16]^ The included T1D GWAS data had a total sample size of 18,942 T1D cases and 501,638 controls of European ancestor (for a detailed description of the T1D GWAS data, see the study by Chiou et al^[17]^). The statistical data for both exposure and outcome were drawn exclusively from European populations to minimize potential bias in the MR analysis due to population stratification (Table 1). As this study utilized only open-source summarized GWAS statistical data, and no attempts were made to identify individual-level data, ethical approval was not required.

**Table 1:** Exposure and outcome GWAS data sources.

Exposure/outcome	Year	Population	Sample size	GWAS ID
White blood cell count	2020	European	563,946	ieu-b-30
Monocyte cell count	2020	European	563,946	ieu-b-31
Lymphocyte cell count	2020	European	563,946	ieu-b-32
Neutrophil cell count	2020	European	563,946	ieu-b-34
Basophil cell count	2020	European	563,946	ieu-b-29
Eosinophil cell count	2020	European	563,946	ieu-b-33
731 Lymphocyte subtypes	2020	European	3757	GCST0001391-GCST0002121
Type 1 diabetes	2021	European	520,580	ebi-a-GCST90014023

### 2.3. Selection of IVs

The single nucleotide polymorphisms (SNPs) with a genome-wide significance, associated with exposure factors variables were selected as IVs.

For WBC count, 5 specific types of WBC count and T1D, the *P*-value parameter was set to 5 × 10^−8^ for identifying IVs. In accordance with recent research,^[15,18]^ the significance level of IVs for each immune trait was set to 1 × 10^−5^. Linkage disequilibrium between SNPs was assessed using the clumping method (*r*^2^ < 0.001, window size = 10,000 kb) on European samples sourced from the 1000 Genomes Project.^[19]^

For each variant included in the genetic instruments, variance (*R*^2^) was calculated using the formula *R*^2^ = 2 × MAF × (1 − MAF) × beta^2^ (where MAF represents the effect allele frequency and beta represents the effect estimate of the genetic variant in the exposure GWAS). *F*-statistics were then calculated for each variant using the formula *F* = *R*^2^(*N* − 2)/1 − *R*^2^ and preserved SNPs with an *F*-statistics > 10 (where *R*^2^ represents the variance in exposure explained by the genetic variant, and *N* represents the number of individuals in the exposure GWAS) to assess the strength of the selected instruments.

In previous Mendelian randomized and clinical observational studies, we found that hypothyroidism, nonalcoholic fatty liver disease, and ketoacidosis have causal relationships with T1D. In order to meet the third hypothesis of Mendelian genetic law, we removed SNPs that have suggestive associations with hypothyroidism, nonalcoholic fatty liver disease, and ketoacidosis (*P* < 5 × 10^−5^).^[20–22]^

### 2.4. Statistical analysis

We utilized the 2-sample MR R software package for MR analysis. The multiplicative random-effects IVW method was used as the primary MR analytical approach. This classical method of MR analysis can estimate IVW meta-analyses of the Wald ratio for each SNP on the outcome and can provide an accurate estimate of the causal effect when all SNPs are valid IVs.^[23]^ In addition, “MR-Egger,” “Weighted Median” and “Weighted Mode” were used as complementary approaches to increase the stability of results.

To further validate the robustness of our results, we undertook several sensitivity analyses. These included a heterogeneity assessment using Cochran Q statistic, horizontal pleiotropy detection via the intercept in MR-Egger regression, as well as a leave-one-out analysis to evaluate the influence of individual SNPs.

MR associative analyses were adjusted for multiple testing by calculating false discovery rate (FDR)-corrected *P* values with the Benjamini–Hochberg method.

A threshold of 0.05 was set to declare significant association for *P*_FDR_ value.

All analyses were performed in R 3.5.3 software.

## 3. Results

### 3.1. Causal estimates between peripheral immune cell count and T1D

After the selection and harmonization of IVs, we utilized 461 SNPs for LYM cell count, 184 SNPs for BAS cell count, 438 SNPs for WBC count, 399 SNPs for EOS cell count, 470 SNPs for MON cell count, 388 SNPs for NEU cell count for MR analysis. All SNPs had *F*-statistics above 10, demonstrating their suitability as strong instruments. The harmonized data are presented in Tables S1 to S12, Supplemental Digital Content, http://links.lww.com/MD/N650

**Figure 2.**
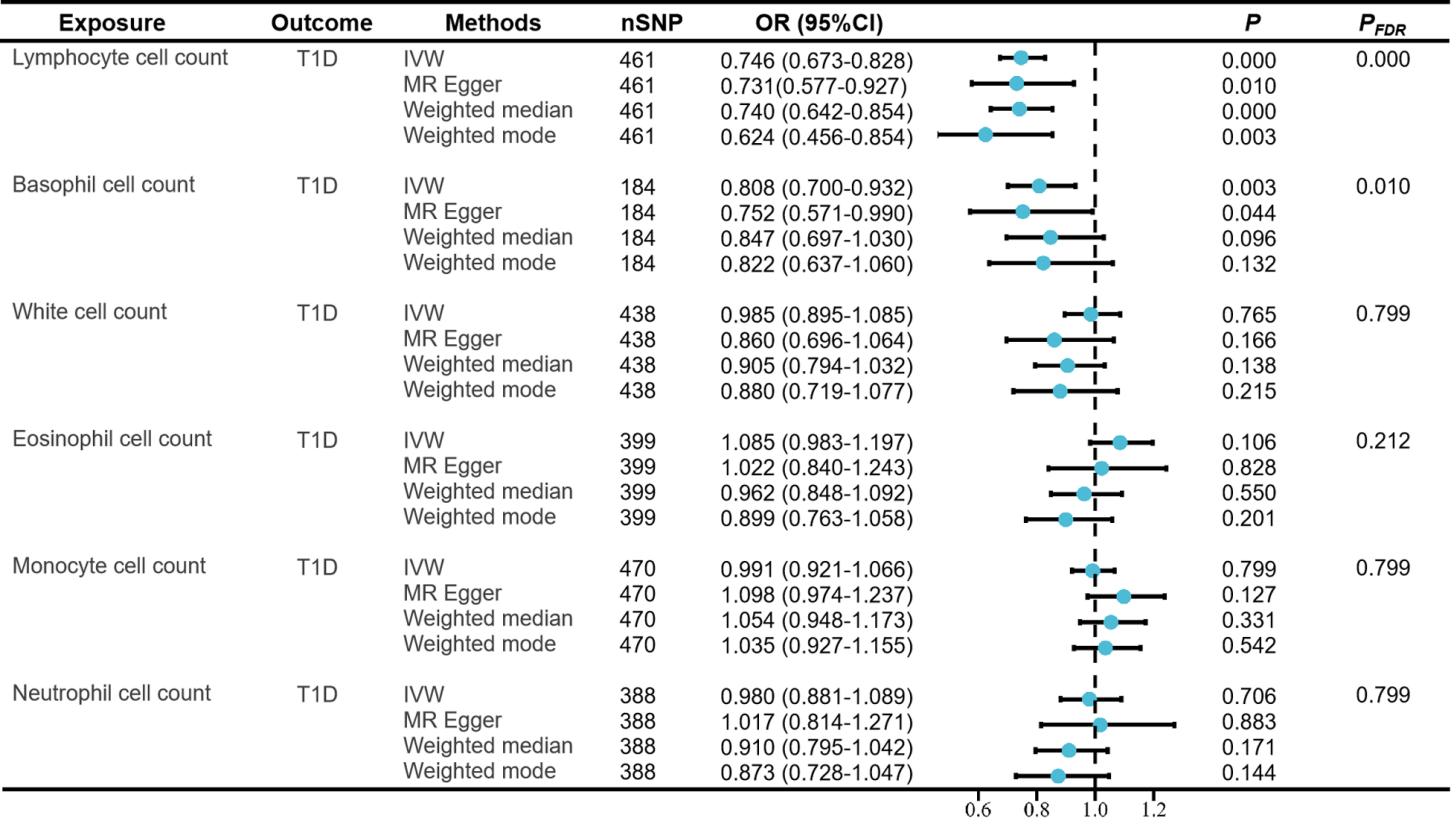
Mendelian randomization estimates of the association between circulating cell count and risk of type 1 diabetes. CI = confidence interval, IVW = inverse variance weighted, OR = odds ratio.

The MR estimation values of the different methods are shown in Figure 2.

Notably, our results demonstrated a strong causal association between an increased liability of T1D and an lower level of circulating LYM cell count (odds ratio [OR] per 1 standard deviation [SD] decrease = 0.746, 95% confidence interval (CI): 0.673–0.828, *P*_FDR_ = 0.036), and BAS cell count (OR per 1 SD decrease = 0.808, 95% CI: 0.700–0.932, *P*_FDR_ = 0.010) using IVW analysis. However, no obvious association was observed between WBC, NEU, MON, or EOS cell count and T1D susceptibility.

**Figure 3.**
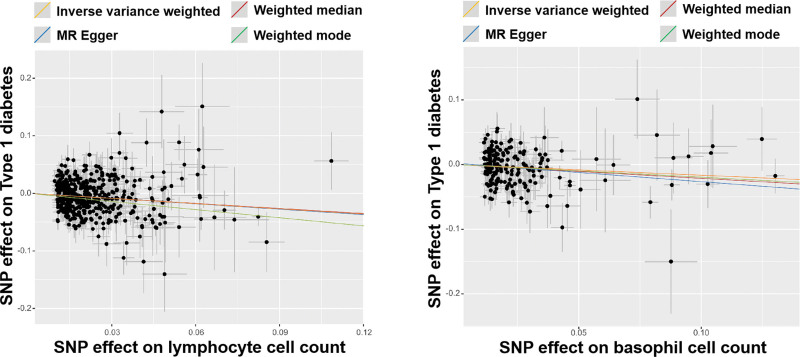
Scatterplot of potential effects of SNPs on lymphocyte and basophil cell count versus type 1 diabetes. The *x*-axis represents the effect of each SNP on lymphocyte and basophil cell count. The *y*-axis represents the effect of each SNP on type 1 diabetes. The gray line around the black solid point indicates the corresponding 95% confidence interval for the effect. The slopes of the solid lines show the effect estimates of the 4 MR methods. MR = Mendelian randomization, SNP = single nucleotide polymorphism.

The estimated effect sizes of SNPs on LYM and BAS cell count and T1D are shown as scatter plots (Fig. 3). The selected WBC count SNP effects (*x*-axis) and T1D SNP effects (*y*-axis) were plotted, and the results showed a negative causal effect on the effect of LYM and BAS cell count and the occurrence of T1D.

**Figure 4.**
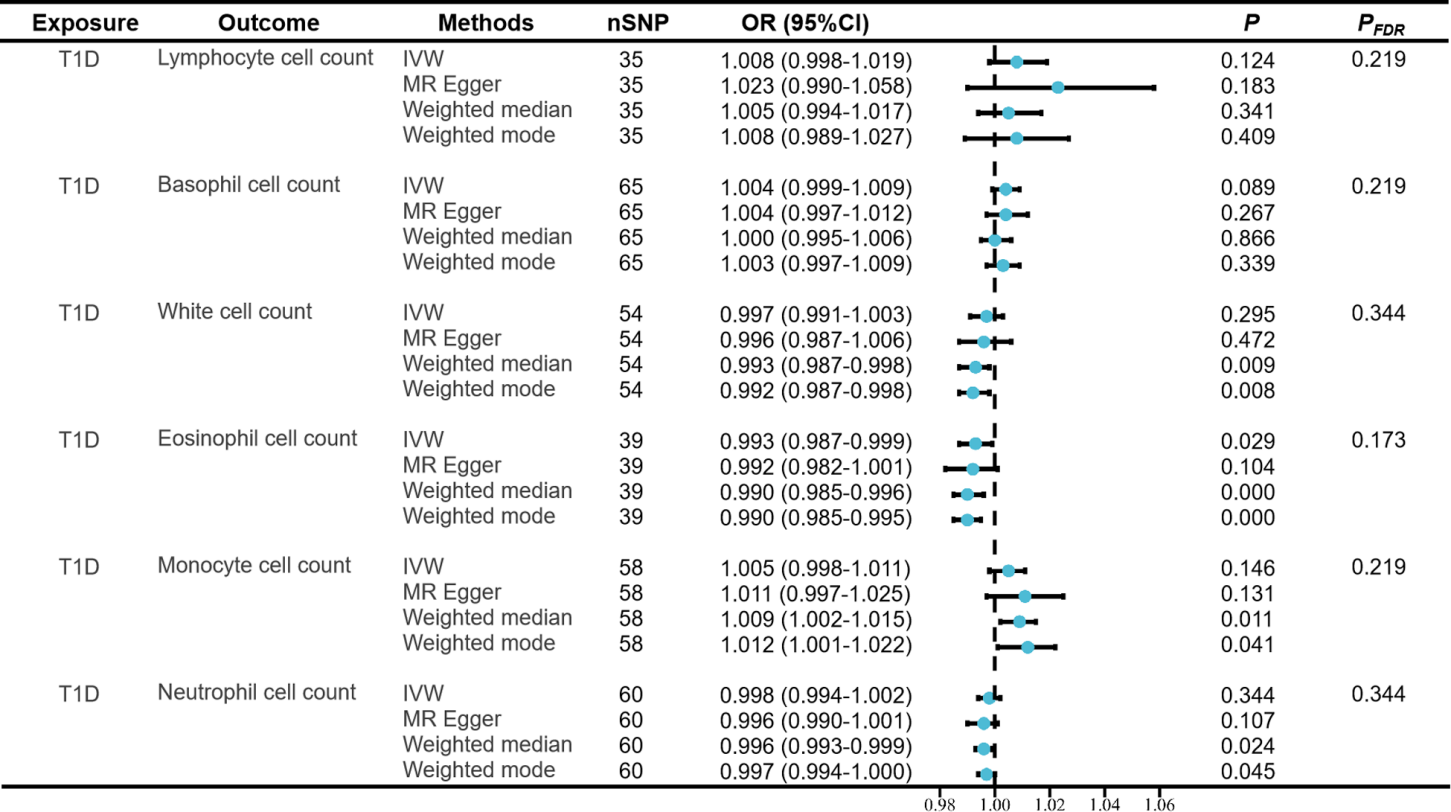
Reverse Mendelian randomization estimates of the association between circulating cell count and risk of type 1 diabetes. CI = confidence interval, IVW = inverse variance weighted, OR = odds ratio.

Next, we performed backward MR analyses to avoid the reverse causality between LYM and BAS cell count and T1D. In the backward MR analyses, T1D was set as exposure and immune cells as outcome. As shown as Figure 4, after FDR, the results showed that no reverse causality was found between T1D and circulating immune cell count.

### 3.2. MR analysis on lymphocyte subtypes cell count with risk of T1D

To test whether lymphocyte subtypes are causally associated with T1D risk, we further carried out MR analysis between T1D and 731 lymphocyte subpopulations.

After FDR adjustment, we detected protective effects of 3 immuno-phenotypes on T1D: CCR2 on plasmacytoid dendritic cell (OR = 0.873, 95% CI: 0.818–0.933, *P*_FDR_ = 0.007), CCR2 on CD62L + plasmacytoid dendritic cell (OR = 0.873, 95% CI: 0.818–0.933, *P*_FDR_ = 0.007), FSC-A on Natural Killer T (OR = 0.705, 95% CI: 0.597–0.834, *P*_FDR_ = 0.007) (Table S13, Supplemental Digital Content, http://links.lww.com/MD/N650). However, no significant association was found between absolute count of lymphocyte subtypes cell and T1D risk (Table S14, Supplemental Digital Content, http://links.lww.com/MD/N650).

### 3.3. Sensitivity analysis

The present study used the MR-Egger intercept test to examine potential pleiotropy.

When monocyte cell count were exposure, the level pleiotropy results showed that *P* = .034, other results revealed that the MR-Egger intercept did not significantly deviate from zero (*P* > .05), indicating the absence of pleiotropy. We also performed a heterogeneity test and computed the I2 statistic, which ranged from 0 to 50, indicating insignificant heterogeneity (Tables S15 and S16, Supplemental Digital Content, http://links.lww.com/MD/N650).

Sensitivity analysis of the IVW results based on the leave-one-out method did not suggest any SNP in IV that had a strong effect on the results, indicating that the effect OR value obtained using the IVW method was relatively robust (Tables S17 to S28, Supplemental Digital Content, http://links.lww.com/MD/N650).

## 4. Discussion

The MR analysis has been commonly applied to elucidate the potential causal relationship between risk factors and diseases. In this study, we used MR analysis to evaluate the role of circulating immune cell count in T1D susceptibility and identified a causal association between LYM and BAS cell count and T1D risk based on GWAS, enriching the existing framework for genetic variants that predispose to autoimmune disease.

T1D is regarded as an organ-specific autoimmune disease, during which lymphocyte cells are involved in a dynamic progression of inflammation of the islet contributing to the loss of pancreatic β cells.^[5]^ For instance, self-reactive CD4+/CD8+ T cells can undergo metabolic reprogramming, leading to pancreatic β-cell damage.^[24,25]^ Regulatory T cells can suppress effector T cells or indirectly inhibit the maturation of antigen-presenting cells by releasing cytokines, such as interleukin (IL)-10 and transforming growth factor-β, thereby shielding pancreatic beta cells from attack.^[26–28]^ Also, B cell deletion can prevent or reverse autoimmune diabetes in nonobese diabetic mice and even result in partially remaining β cell function in patients with new-onset T1D.^[29]^ At present, it is unclear whether lymphocyte count is related to the occurrence of T1D. Herein, we first found suggestive evidence that each SD decrease in LYM count corresponded with an elevated risk of T1D. The counted cells were circulating LYM cells, and we did not consider infiltrating the pancreatic islets, which differs from previous studies. We further investigated the relationship between the count of lymphocyte subtypes and T1D by MR analysis. Regrettably, after FDR correction, no statistical difference was found.

BAS cell stem from CD34+ hematopoietic progenitor cells in the bone marrow and was released into circulation as mature cells.^[30]^ Harsunen et al^[10]^ demonstrated that people who develop T1D have decreased numbers of BAS cell at and prior to the diagnosis of T1D. The causal link between BAS cell count and T1D is unknown, as the observed associations cannot exclude reverse causation. This study explored the causal relationship between circulating cell count and T1D at the genetic level and found that a lower BAS cell count was a risk factor for T1D. The physiological role of BAS cell in immune responses is enigmatic, although it has been observed for many years, and some studies have reported that BAS cell has a negative regulatory role in immune responses.^[31,32]^ Previous research showed that the destruction of β cells in T1D is driven by IFNγ release from Th1 cells, and as IL-4 counter regulates Th1 responses and has been shown to improve Th1-driven autoimmune diseases.^[33,34]^ Also, Hübner et al demonstrated that chronic BAS cell activation could protect against T1D by release of IL-4.^[35]^ Moreover, histamine, which is released from BAS cell, has been shown in vitro to suppress Th1 responses by signaling through the H2 receptor on lymphocytes. However, the full impact of BAS cell count on the pathogenesis of T1D and their potential as a therapeutic target requires further research.

Previous observational studies have frequently shown decreased WBC and NEU cell count in patients with T1D compared with healthy controls.^[36,37]^ Furthermore, Valle et al ^[38]^ identified a decline in peripheral blood NEU cell count preceding the onset of T1D. However, our MR analysis did not find a significant causal relationship between WBC and NEU cell count and T1D.

The strength of our study was its ability to overcome potential confounding factors present in observational studies, such as clinical heterogeneity arising from disease duration, sample quality, microbiota composition, clinical interventions, and diagnostic criteria. The large sample size ensured greater consistency in the obtained results, making it feasible to evaluate causal effects.

This study had some limitations. First, we relied on GWAS data without access to individual data and without conducting a stratification analysis. Consequently, we were unable to explore the causal relationship between circulating blood cell count and the incidence of T1D in patients of varying onset times and ages. Second, GWAS data were derived from a single racial group. The generalizability of these findings should be confirmed by incorporating a larger sample size and extending the analysis to include more racial groups. Lastly, we used peripheral blood cell count, which cannot reflect the functionality of various cells or their infiltration into pancreatic islets.

## 5. Conclusion

To summarize, this study provides evidence that a lower LYM and BAS cell count increases the risk of T1D. Further mechanistic understanding of the changes in peripheral immune cell numbers will shed light on the etiology of T1D and provide targets for intervention for prevention and treatment.

## Acknowledgments

We thank the UK Biobank consortium, Blood Cell Consortium and Chiou, et al for providing GWAS summary statistics data for our analysis. In addition, we thank Dr Yuan Cai (The Chinese University of Hong Kong) for her assistance with the statistical analysis and Ms Jing Lin (Beijing Tiantan Hospital) for her support. This study was supported in part by the Advanced Innovation Center for Human Brain Protection.

## Author contributions

**Data curation:** Jing Wang, Yukun Xiang, Ningning Wang, Xin Zhao, GengYan Liu.

**Formal analysis:** Jia Luo, Jing Wang.

**Methodology:** Jia Luo.

**Supervision:** Lihui Liu, Haoxiao Chang.

**Writing – original draft:** Jia Luo.

**Writing – review & editing:** Lihui Liu, Haoxiao Chang.

## Supplementary Material


